# Individually tailored physiotherapy interventions for pregnancy-related pelvic girdle pain: functioning, physical activity, and treatment satisfaction four months postpartum. A cross-sectional study

**DOI:** 10.1186/s12884-026-09617-w

**Published:** 2026-07-07

**Authors:** Annika Svahn Ekdahl, Monika Fagevik Olsén, Annelie Gutke

**Affiliations:** 1 Department of Health and Rehabilitation, Sahlgrenska Academy, University of Gothenburg, Box 455, Gothenburg, SE-405 30 Sweden; 2 Department of Physiotherapy, Sahlgrenska University Hospital, Gothenburg, Sweden; 3 Region Västra Götaland, Närhälsan Eriksberg, Gothenburg, Sweden

**Keywords:** Individually tailored, Pelvic girdle pain, Physiotherapy, Postpartum follow-up, Pregnancy

## Abstract

**Background:**

Pregnancy-related pelvic girdle pain (PPGP) affects more than 50% of pregnant women, and for some it may lead to persistent postpartum pain. The factors affecting the prevalence of PPGP postpartum are unclear. Recommended interventions for PPGP include exercise, pelvic belts, acupuncture, and promotion of physical activity. However, individually tailored interventions based on treatment guidelines are necessary given the multifaceted nature of PPGP, but this has not been evaluated in a clinical setting. The aim of this study was to describe levels of functioning, physical activity, pain, and treatment satisfaction at four months postpartum in women who received individually tailored physiotherapy based on treatment guidelines for PPGP and to investigate relationships between factors that could impact disability resulting from PPGP postpartum.

**Materials and methods:**

A cross-sectional study based on questionnaires answered four months postpartum by 164 women who received physiotherapy for PPGP during pregnancy. Treatment satisfaction, and functioning measured by the Pelvic Girdle Questionnaire (PGQ), were the primary outcomes, and multiple linear regression analyses were performed to investigate factors with a possible impact on the PGQ score.

**Results:**

Of the total respondents (*n* = 164), 125 women reported (76%) reported PPGP four months postpartum, of whom 40% (*n* = 50) experienced moderate-to-severe disability according to the PGQ. Most women were satisfied with the physiotherapy treatment, regardless of their level of disability. Concern about pain was the only variable associated with the PGQ score. Despite limitations in functioning among women who reported pain, one third reached the recommended levels of physical activity.

**Conclusion:**

Although 76% of women experienced persistent PPGP four months postpartum, individually tailored physiotherapy for PPGP during pregnancy was perceived as an intervention with positive impact. Concern about pain appears to be associated with postpartum functioning, but the study design precludes causal conclusions.

**Trial registration:**

FoU in Region Västra Götaland, Sweden No. 220,091, https://www.researchweb.org/is/vgr/project/220091.

**Supplementary Information:**

The online version contains supplementary material available at 10.1186/s12884-026-09617-w.

## Background

Pregnancy-related pelvic girdle pain (PPGP) is a common condition that may substantially interfere with a woman’s daily functioning, both during and after pregnancy, encompassing all domains of the International Classification of Functioning, Disability and Health (ICF) [1]. This condition affects more than 50% [2–4] of pregnant women, limits functioning, and negatively affects women’s ability to manage work, social life, and physical activity [5]. Feeling limited and less independent may result in emotional distress; depressive symptoms during pregnancy and/or postpartum are more common among women with PPGP than among healthy pregnant women [6, 7]. Pregnant women who experience PPGP have an increased risk of sick leave [8], and PPGP is, after pregnancy complications, one of the most common reasons for sick leave during pregnancy [9].

The experience of pain involving the sacroiliac joints and/or the pubic bone varies greatly among individuals, as pain may occur at any gestational age or within weeks after delivery [10]. For most women, symptoms disappear within three months postpartum [11, 12], but approximately 25% experience pain 3–6 months after delivery [12, 13], and for 10% of these women persistent pain for up to 11 years has been reported [14]. Risk factors for postpartum PPGP include lumbopelvic pain before pregnancy, and during pregnancy, a body mass index (BMI) > 25 kg/m^2^, severe pain, heavy work or uncomfortable working positions, depressive symptoms and/or catastrophizing and fear-avoidance beliefs [15, 16].

The etiology of PPGP remains unclear but is generally regarded as multifactorial, with biomechanical, hormonal, inflammatory, and psychological factors proposed as potential contributors [17, 18]. Recommended interventions for PPGP include exercise to improve muscle function in the trunk, pelvis and hips; a pelvic belt; acupuncture; and promoting maintenance of physical activity [10, 19, 20]. The latter, during pregnancy, may reduce the risk of complications such as hypertension, diabetes, and preeclampsia [21] and can also prevent reduced muscle function in the trunk and hips, a factor that may influence disability for women with postpartum PPGP [22].

Women who experience PPGP during pregnancy often seek advice and treatment adapted to their individual situation, as many wish to remain independent, physically active, and able to manage everyday activities [23, 24]. However, both pain and a lack of knowledge regarding symptoms and safe activities have been reported as barriers to maintaining physical activity [5]. Managing pain is therefore considered an important aspect of care [23]. Interventions such as acupuncture or transcutaneous electrical nerve stimulation have been shown to reduce pain intensity and pain-related concern and may help preserve physical activity levels [25]. Although such interventions primarily address PPGP from a biomedical perspective, they may not capture the full complexity of the condition. Pregnancy-related PPGP can affect women’s functioning in several aspects of daily life and necessitates a biopsychosocial approach to management [26]. Because of the multifaceted presentation, treatment strategies should be adapted to each woman’s individual needs and circumstances [18]. If women with PPGP receive individually tailored interventions, risk factors for postpartum pain may be identified and addressed, potentially preventing persistent pain [15]. A person-centered physiotherapy approach based on existing treatment guidelines for pregnancy-related PPGP [10, 19, 20], may therefore be relevant in clinical practice. However, such physiotherapy interventions have not previously been evaluated in an ordinary clinical setting. Consequently, it remains unclear whether pregnant women with PPGP are satisfied with the interventions provided or whether factors related to PPGP influence functioning four months postpartum.

### Aims

This study, conducted four months postpartum among women who had received individually tailored physiotherapy for PPGP during their most recent pregnancy, had three specific objectives:

- assess satisfaction with the physiotherapy received,
- describe functioning, physical activity, and pain four months postpartum, and
- examine associations between factors that may influence postpartum functioning.

## Methods

### Design

This cross-sectional questionnaire-based study, conducted four months postpartum, adhered to the Declaration of Helsinki, was approved by the Regional Ethical Review board in Gothenburg, Sweden (No. 105 − 16) and registered in FoU in Region Västra Götaland No. 220,091, https://www.researchweb.org/is/vgr/project/220091.

The study was reported using the Strengthening the Reporting of Observational Studies in Epidemiology (STROBE) statement for cross-sectional studies [27].

### Setting and participants

Participants were recruited through a physiotherapist, specialized in women’s health, at a primary health care center in southwestern Sweden. Recruitment was conducted 2017–2021 from the consecutive flow of the clinic; no advertising was made. An independent researcher obtained contact information of eligible participants, established a code list containing participant identification numbers which was used consistently throughout the study, and distributed the questionnaire by mail together with written information about the study purpose, voluntary participation, and data handling. Contact details to the research team were provided so that participants could ask questions if needed. Signed informed consent was obtained on a separate consent form that was returned together with the completed questionnaire. One initial mailing was made to 302 women who received physiotherapy for PPGP during their most recent pregnancy. One reminder was sent after three weeks to non-responders, plus a second reminder after additionally one week if needed.

#### Inclusion criteria

This study included women, four months postpartum, who had received physiotherapy for clinically verified PPGP during their most recent pregnancy.

#### Exclusion criteria

Insufficient proficiency in Swedish to understand the study information, provide informed consent, and complete the questionnaire; major obstetric or neonatal complications (e.g., miscarriage, stillbirth, preterm birth, or serious neonatal illness); systemic diseases affecting the musculoskeletal system (e.g., rheumatologic or neurological disorders); and fractures or surgical procedures involving the pelvis or spine.

### Measurements

The questionnaire used in this study was developed in Swedish and included both validated instruments and additional questions developed by the research team (Additional file 1). The primary outcomes at four months postpartum were treatment satisfaction and self-reported functioning measured using the Pelvic Girdle Questionnaire (PGQ) [28]. The PGQ total score (PGQ-T) was used to classify disability levels among women reporting PPGP four months postpartum as low (< 28), moderate (28–61), or severe (≥ 62) disability [29]. Other measurements included physical activity level, pain intensity and localization, general health, pain-related concern, and perceived recovery (Table 1).

**Table 1:** Outcome measures

Outcome	Measurement	Information
Treatment satisfaction	Numeric Rating Scale (NRS) of 0–10; 10 = completely satisfied	Perceived satisfaction regarding received treatment.
Functioning	Pelvic Girdle Questionnaire (PGQ)* [28, 30] 0% = no disability	A total score (PGQ-T) + scores for the subscales of “Activity” (PGQ-A) and “Symptoms” (PGQ-S).
Physical Activity	Two single-item questions [31]	Number of days/week with moderate intensity activity for 30 min and number of days/week with high intensity activity for 20 min was recorded. Summarized into yes/no for fulfilling activity recommendations of 150 min/week [32]
Pain intensity	Two NRS of 0–10; 10 = worst pain [33]	Pelvic girdle pain on average + pain at its worst during the last 48 h.
Pain localization	Pain drawing [34]	The pain location was marked on a female body chart, and the number of locations was recorded.
Health	NRS of 0–10; 0 = best imaginable health	Perceived current overall health status marked on a horizontal NRS.
Concern about pain	NRS of 0–10; 10 = extremely worried [35]	Current concern about pain.
Recovery	Global Rating of Change (GRC) of 0–10; 0 = very much worse, 5 = unchanged, 10 = completely recovered [36]	Perceived change of symptoms since physiotherapy contact.

* After completion of data collection, we were informed that the Swedish version of PGQ [30] differs from the original questionnaire [28] regarding one item. In the Swedish version, the timeframe in item 8 is 10 min whereas it is 60 min in the original version

Descriptive background variables collected included age, parity, BMI, marital status, education level, socioeconomic status, and previous health issues or diagnoses. Additional questions addressed pain in the lower back/pelvic girdle before pregnancy, delivery mode and potential complications, and possible side effects of the physiotherapy treatment. Participants were also given the opportunity to provide written comments regarding barriers to physical activity, treatment side effects, and any perceived unmet needs in the physiotherapy intervention.

### Physiotherapy interventions

Interventions took place at one primary care rehabilitation clinic and were based on current guidelines [10, 19, 20], adapted to the women’s individual needs through a process of clinical reasoning and shared decision-making between the physiotherapist and the individual woman; thus, the number of sessions varied. During the initial 45-minute session, a comprehensive anamnesis was conducted to explore the woman’s medical history, assess the impact of PPGP on her daily activities, and evaluate her level of understanding regarding the condition. A systematic clinical examination [37] was performed to differentiate PPGP from lumbar pain. Classification of PPGP was made if the onset of pain was pregnancy-related, pain localised between the posterior iliac crest and gluteal fold and/or over the pubic symphysis, with or without radiation into the thigh, and reproducible by two or more pelvic pain provocation tests [37].

All participants received individually tailored advice on managing daily activities and work-related tasks, as well as strategies for engaging in physical activity. While similar advice could be provided to participants with comparable symptoms or challenges, the guidance was adapted to each individual’s situation. Participants were also given the opportunity to ask questions about their symptoms. Most women were offered a fitting of a pelvic belt for pain management and to facilitate activity, along with instructions for pelvic floor muscle training and a few general exercises targeting the trunk and hip muscles. A 30-minute follow-up visit was scheduled to assess progress and adjust advice and/or exercises as needed (e.g., if they were ineffective, aggravated pain, or were considered too easy). All participants were offered a minimum of two visits. Depending on individual needs, treatment response, and the time remaining until delivery, some women received additional 30-minute sessions. These included specific interventions such as acupuncture, transcutaneous electrical nerve stimulation (TENS), or targeted strengthening exercises with or without external resistance (e.g., elastic bands or weights) for the affected muscle groups.

### Analysis

Data were analyzed using SPSS version 28 (IBM Corp., Armonk, NY, USA). Descriptive statistics were calculated for all variables and presented as mean (standard deviation, SD) or number (percentage). Group differences were examined using the Pearson chi² test for categorical variables and the independent t-test or one-way ANOVA for continuous variables when comparing two or three groups, respectively. P-values < 0.05 were considered statistically significant. As the study had an exploratory aim, no correction for multiple testing was performed, except for post-hoc tests conducted when ANOVA or Pearson chi² tests revealed significant group differences, for which Bonferroni correction was applied.

Free-text responses regarding treatment satisfaction and reasons for not being as physically active as desired were reviewed and grouped into categories by content. The number of responses within each category was then counted and summarized descriptively.

Multiple linear regression analysis was performed with the PGQ-T (continuous scale) as the dependent variable. Previous studies have investigated the prevalence of postpartum pain and potential risk factors [38], but to our knowledge, the degree of disability measured with the PGQ in relation to these risk factors has not been investigated. Therefore, a full-model approach was used including the following independent variables: age, parity, delivery mode, concern about pain, and experience of low back pain and/or pelvic girdle pain before pregnancy [15, 16, 38, 39]. Pain intensity (NRS) was not included because pain is already incorporated in the PGQ-T. Overall health (NRS) was also excluded because the measure reflects not only PPGP but also other health conditions (e.g., other diagnoses, sleep disorders, depressive symptoms, stressful life events), which may reduce its specificity in relation to PPGP. BMI was not included due to the natural changes in body weight during and shortly after pregnancy, and physical activity level was excluded because PPGP itself may limit activity. Education level was not included as most participants had a higher level of education.

A second regression analysis was performed with the PGQ activity subscale (PGQ-A, continuous scale) as the dependent variable to investigate associations between the independent variables listed above and activity limitations, excluding the symptom subscale (PGQ-S).

The assumptions of multiple linear regression were evaluated by examining residuals for normality, homoscedasticity, and independence, and by assessing multicollinearity among the independent variables. All assumptions were considered to be met.

## Results

The participants answered the survey according to their latest pregnancy; 164/302 surveys (54%) were returned. The mean age was 32.6 years, 99% were married/cohabiting, and most women had completed higher education and reported that they managed well financially. Of the total respondents (*n* = 164), 125 women (76%) reported PPGP four months postpartum. Only four women reported severe disability on the PGQ-T; therefore, the participants were dichotomized into two groups: PGQ-T < 28 (low disability) and PGQ-T ≥ 28 (moderate-to-severe disability). The proportion of primiparous women was higher among women without pain (63%) than among those with moderate-to-severe disability (42%) (Table 2). Vaginal delivery was most common, and approximately 27% of the women reported delivery complications in free-text comments, including major bleeding and perineal tears; the degree of perineal tear was not specified. There were no statistically significant differences in any of the descriptive variables, except for Global Rating of Change (GRC), between women without pain, and those with low (< 28) or moderate-to-severe disability (≥ 28) according to the PGQ-T. Women without pain or with low disability, according to the PGQ-T, reported a more favorable change in their condition at four months postpartum compared to when they sought physiotherapy for their complaints during pregnancy (*p* < 0.001 for all three comparisons between groups) (Table 2).

**Table 2:** Descriptive data four months postpartum for participants without pain (*n* = 39), and with self-reported PPGP (*n* = 125)

		With self-reported PPGP, *n* = 125	
Variable	**No pain**, *n* = 39	**PGQ-T < 28**, *n* = 72*	**PGQ-T ≥ 28**, *n* = 51
Age in years, mean (SD)	31.8 (3.68)	32.5 (4.03)	33.4 (4.40)
BMI, mean (SD)	25.3 (4.45) *n* = 38	25.5 (4.77)	26.3 (4.34)
Parity, *n* (%)			
1	24 (63.2)	38 (52.8)	21 (42.0)
≥ 2	14 (36.8) *n* = 38	34 (47.2)	29 (58.0) *n* = 50
Married/cohabiting, *n* (%)	38 (97.4)	71 (100) *n* = 71	51 (100)
Education level, *n* (%)			
Elementary/High school	8 (20.5)	11 (15.3)	14 (27.5)
College/University	31 (79.5)	61 (84.7)	37 (72.5)
Manage financially; yes, *n* (%)	35 (89.7)	66 (91.7)	43 (84.3)
Previous diagnosis; yes, *n* (%)	8 (20.5)	14 (19.4)	11 (22.0) *n* = 50
Vaginal delivery; yes, *n* (%)	30 (76.9)	55 (76.4)	43 (86.0) *n* = 50
Complications during delivery; yes, *n* (%)	10 (25.6)	19 (26.4)	14 (27.5)
Reaches recommended PA; yes, *n* (%)	26 (66.7) *n* = 38	20 (27.8)	19 (37.3) ** *n* = 50
Overall health status, NRS, mean (SD) 0 = best imaginable health	3.6 (3.06)	4.2 (2.44)	4.9 (2.39) *n* = 50
Global rating of change, GRC, mean (SD) 10 = totally recovered	9.7 (0.51) *n* = 37	8.4 (1.28)	6.9 (1.84) ***
Treatment satisfaction, NRS, mean (SD) 10 = completely satisfied	8.8 (2.31) *n* = 37	8.2 (2.39)	7.9 (2.67) *n* = 50

Participants with self-reported PGP were dichotomized into two groups; PGQ-T score <28 (low disability) and ≥28 (moderate-to-severe disability)

*PPGP* Pregnancy-related pelvic girdle pain, *PGQ-T* Pelvic Girdle Questionnaire total score, *SD* standard deviation, *BMI* body mass index (kg/m^2^), *NRS* numeric rating scale 0-10, *PA* physical activity, *GRC* Global Rating of Change Scale: Perceived change in symptoms since physiotherapy

* Two participants excluded due to missing items on the PGQ-T

**Post-hoc test revealed significant differences (Bonferroni corrected, *p*<0,017) between groups a) no pain and PGP <28 and b) no pain and PGQ ≥28

***Post-hoc test, Tukey HSD, revealed significant differences (*p*<0,001) in all three comparisons between groups

**Fig. 1:**
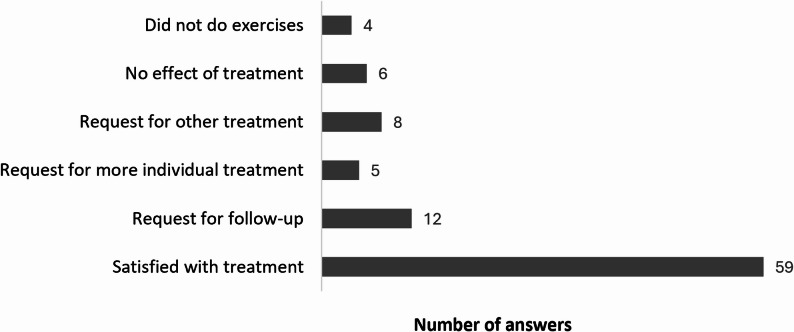
Free-text responses on perceived gaps in pelvic girdle pain treatment. Categorized free-text responses from 94 participants to the question of whether anything was missing from the treatment they received for PPGP. Bars represent the number of responses per category.

Approximately 20% of the participants reported having a previous diagnosis. The most common reported diagnoses were asthma, hypothyroidism, depressive symptoms, and migraine. Overall, most women were satisfied with the treatment and advice provided by the physiotherapist (Table 2). In response to the questionnaire item asking whether any aspects of PPGP treatment were lacking, 94 women (75%) provided free-text comments. Among these, 58 women reported no unmet needs and expressed satisfaction with the treatment received. Twelve women indicated a need for additional follow-up during the remainder of pregnancy and/or the postpartum period. Other comments highlighted challenges in adhering to recommended exercises, commonly attributed to self-reported lack of discipline or motivation, and requests for additional interventions, including water aerobics, massage, care for vaginal prolapse, and referral for psychological support (Fig. 1).

**Fig. 2:**
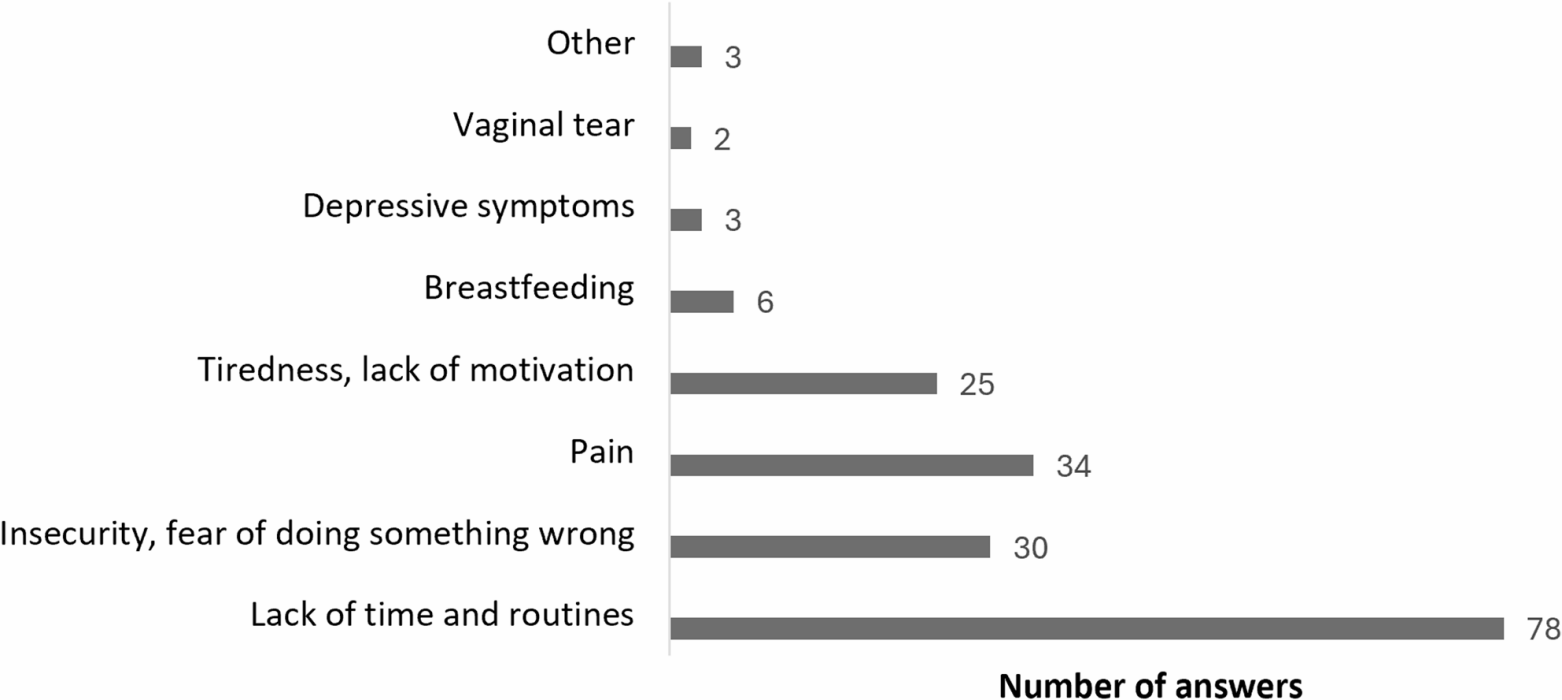
Free-text responses on barriers to physical activity postpartum. Categorized free-text responses from all participants (*n* = 164) to the question of why they were not as physically active as they wanted. Bars represent the number of responses per category; participants could report more than one reason.

Women who reported PPGP four months postpartum, regardless of disability level measured by the PGQ, were significantly less physically active (*p* < 0.017) than women who reported no pain (Table 2). Among women who were less physically active than desired, the most reported barrier was lack of time or routines (*n* = 78). Other frequently reported reasons included pain (*n* = 34), uncertainty or fear of doing something wrong (*n* = 30), and tiredness or lack of motivation (*n* = 25). Less frequently reported reasons included breastfeeding, depressive symptoms, vaginal prolapse, and other individual factors (Fig. 2).

Analgesic use was low, and half of the women with postpartum pain had experienced low back pain and/or pelvic girdle pain before their latest pregnancy. The subgroup analysis comparing women with low disability to those with medium/high disability revealed significant differences for the PGQ-T (*p* < 0.001) and the subscales PGQ-A and PGQ-S (*p* < 0.001, respectively). No differences were detected between the subgroups regarding pre-pregnancy low back or pelvic girdle pain (Table 3). 

**Table 3:** Symptom characteristics among women (*n* = 125) with self-reported PPGP four months postpartum

Variable	PGQ-T < 28, *n* = 72*	PGQ-T ≥ 28, *n* = 51	*p*-value
Pain affects daily activities; yes, *n* (%)	17 (22.7)	32 (64.0)	*< 0.001*
Pain how often, *n* (%)			*<0.001*
Some days	55 (77.5)	16 (31.4)	
Most days	13 (18.3)	16 (31.4)	
Every day	3 (4.2)*n* = 71	19 (37.3)	
Pre-pregnancy low back/pelvic girdle pain; yes, *n* (%)	34 (47.2)	26 (51.0)	*0.681*
Average pain, NRS, mean (SD) *0 = no pain*	3.5 (1.99)	5.2 (2.21)	*< 0.001*
Worst pain, NRS, mean (SD) *0 = no pain*	4.3 (2.30)	6.4 (2.21)	*< 0.001*
Concern about pain NRS, mean (SD) *0 = no concern*	2.9 (2.37)	5.4 (2.93)	*< 0.001*
Use of analgesics; yes, n (%)	3 (4.5) *n* = 66	6 (11.8)	*0.125*
PGQ-T, mean (SD)	14.5 (7.90)	44.2 (13.17)	*< 0.001*
PGQ-A, mean (SD)	14.0 (8.00)	42.7 (14.18)	*< 0.001*
PGQ-S, mean (SD)	17.3 (10.65)	46.8 (17.98)	*< 0.001*

Participants with self-reported PGP were dichotomized into two groups; PGQ-T score < 28 (low disability) and ≥ 28 (moderate-to-severe disability)

*PPGP* Pregnancy-related pelvic girdle pain, *SD* standard deviation, *NRS* numeric rating scale 0–10; PGQ, Pelvic Girdle Questionnaire *T* total score, *A* activity score, *S* symptoms score, 0–100, 0 no disability

* Two participants were excluded because of missing items on the PGQ-T

In free-text comments, five participants reported transient treatment-related adverse effects, including back muscle soreness (*n* = 1), discomfort associated with the stabilizing belt (*n* = 2), and exacerbation of PPGP symptoms following examination and/or exercise (*n* = 2).

The multiple linear regression model (Table 4) showed that the set of independent variables was associated with the PGQ-T score at four months postpartum among women reporting PGP (*n* = 125), with an R² of 0.250 (*p* < 0.001). Concern about pain was the only variable significantly associated with the PGQ-T score (B = 3.012, *p* < 0.001). To examine whether this finding was influenced by the symptoms subscale included in the PGQ-T, a second multiple linear regression analysis was performed using the PGQ activity subscale (PGQ-A) as the dependent variable. The independent variables were similarly associated with the PGQ-A score (R² = 0.245, *p* < 0.001), and concern about pain remained the only variable significantly associated with the outcome (B = 2.970, *p* < 0.001).

**Table 4:** Factors associated with PGQ-T among women with self-reported PPGP at four months postpartum

	Multiple linear regression analysis		
Variable	B	95% CI for B	*p*-value
(Intercept)	6.01	-16.52 to 28.54	0.60
Age	0.17	-0.55 to 0.89	0.64
Parity	1.31	-4.65 to 7.26	0.67
Delivery mode	-1.21	-8.55 to 5.93	0.72
Concern	3.01	2.02 to 4.01	< 0.001
Pre-pregnancy low back/ pelvic girdle pain	2.90	-2.85 to 8.64	0.64

Multiple linear regression analysis with the PGQ-T as dependent variable (*n* = 125)

*PGQ-T* Pelvic Girdle Questionnaire total score, *B* Unstandardized regression coefficient, *CI* Confidence interval

As the Swedish version of the PGQ differs from the original [28] in one item, we decided to redo all analyses where the PGQ was used, without item no. 8. These new analyses did not result in any changes to our main result; therefore, we made the decision to keep our original calculations.

## Discussion

The aim of this study was to describe levels of functioning, physical activity, and pain four months postpartum among women who had received individually tailored physiotherapy for PGP during pregnancy, to explore their satisfaction with the treatment received, and to investigate factors associated with postpartum functioning. The main findings show that a substantial proportion of women reported persistent symptoms four months postpartum, with 76% reporting ongoing PPGP and 40% reporting moderate-to-severe disability according to the PGQ. This highlights the importance of continued assessment and management after childbirth. As pain is often experienced recurrently throughout a woman’s life, it may sometimes be perceived as a normal part of being a woman. However, if pain during pregnancy or the postpartum period is not adequately addressed, it may lead to undertreatment and increase the risk of persistent pain [40].

The proportion of women reporting persistent symptoms four months postpartum may appear high. However, all participants in the present study had clinically verified PPGP during pregnancy and had sought physiotherapy treatment, suggesting that they may represent a group with more severe pain and thereby a higher risk for persistent symptoms [16]. Rather than focusing solely on the presence or absence of pain, the present study aimed to explore how functioning was affected postpartum. The use of the PGQ allowed assessment of both pain and its impact on everyday functioning, which is important since women with PPGP often report difficulties managing daily activities because of pain [24, 26, 41]. Research focusing solely on pain intensity or dichotomous measures of PPGP yes/no [13] may therefore underestimate the extent to which the condition affects daily functioning in all domains of the ICF [42]. Since heterogeneity in PPGP measurements has been identified, and different primary outcomes have been used in previous studies, it may be difficult to compare results [43].

Women with lower disability (PGQ-T < 28) reported greater improvements in symptoms than women with higher disability levels. However, changes in symptoms over time may reflect not only recovery from PPGP but also other factors related to pregnancy, childbirth, or life circumstances during the postpartum period.

Although 76% of the women reported persistent symptoms four months postpartum, treatment satisfaction did not differ between women with low disability and those with moderate-to-severe disability. Overall, most women were satisfied with the physiotherapy they had received. This may reflect that satisfaction with healthcare is influenced by factors beyond symptom resolution, such as feeling supported, receiving clear information, and learning strategies to manage symptoms in everyday life [44]. Person-centered physiotherapy approaches that provide individualized advice and reassurance may therefore be perceived as beneficial even when symptoms persist [45].

However, free-text comments provided by 94 women (Fig. 1) indicated a desire for more regular follow-up during the remainder of pregnancy and after childbirth. Similar findings have been reported in studies of PPGP [24, 46] and other musculoskeletal conditions [47]. A postpartum physiotherapy consultation could provide an opportunity to identify treatable or preventable health issues, offer individually tailored advice, and address concerns related to persistent pain and activity limitations [48].

Concern about pain was the only variable with a statistically significant association with the PGQ-T score in the multiple regression analyses among the 125 women who reported PPGP. This finding suggests that psychological factors related to pain perception may play an important role in postpartum functioning, regardless of the degree of physical disability. Pregnant women with PPGP often express concerns about the consequences of pain for their daily life and about whether symptoms will worsen during pregnancy or persist after delivery [24]. Concern about pain may reflect emotional distress, depressive symptoms, or catastrophizing thoughts, which have previously been associated with persistent lumbopelvic pain postpartum [49]. Emotional distress during pregnancy has also been associated with postpartum PPGP [50], and depressive symptoms postpartum are three times more prevalent in pregnant women with lumbar pain/PPGP than in pregnant women without these symptoms [7]. Furthermore, depressive symptoms have been shown to be associated with both the development and intensity of pelvic girdle pain during pregnancy [51]. Psychological characteristics may also influence how pain is perceived and how women recover from PPGP, as personality traits such as higher neuroticism have been linked to greater pain intensity and persistent symptoms, whereas traits such as extraversion and conscientiousness are associated with better recovery [52]. Concern about pain appears to be an important factor in PPGP. However, due to the cross-sectional design, it is not possible to determine whether this concern was present during pregnancy or related to persistent PPGP four months postpartum. Nevertheless, pain-related concern warrants further investigation and may be an important aspect for healthcare professionals to address when managing PPGP during pregnancy and the postpartum period [16]. This is also reflected in recent clinical practice guidelines, which recommend that postpartum management should incorporate both physical and biopsychosocial factors when planning individualized physiotherapy [53].

Approximately one third of the women who reported PPGP four months postpartum met the general recommendations [32] for physical activity irrespective of disability level. This is consistent with previous research on women’s physical activity levels [54], suggesting that persistent symptoms, as well as factors such as tiredness, uncertainty about safe activities, and lack of time and routines as a new parent, influenced physical activity behavior. Lumbar pain and/or PPGP are common reasons for reduced physical activity during pregnancy [55], and sedentary behavior in combination with lumbar pain or PPGP at four months postpartum has been associated with persistent pain at 11 months postpartum among primiparous women [56]. Early interventions focusing on pain management and the promotion of safe physical activity may therefore play an important role in supporting recovery and reducing the risk of pregnancy complications and postpartum depressive symptoms [21, 57].

### Strengths and limitations

The participants were recruited from the consecutive flow of patients attending a primary care rehabilitation clinic located in an urban area, that is representative of a Swedish city. All participants had clinically verified PPGP and were treated by the same physiotherapist who had extensive experience in managing PPGP and was familiar with existing treatment guidelines. While this ensured a consistent knowledge base for treatment, it may also limit the generalizability of the findings, as outcomes could differ in settings with varying levels of expertise or different treatment approaches.

Overall health was measured using a reverse numerical rating scale (NRS) ranging from 0 to 10, where 0 represented the best imaginable health. This format may be less commonly used and may potentially increase the risk of misinterpretation. However, when these results were compared with data from a previous study by our research group that used a conventional NRS format, no substantial deviations were observed.

Although the recruiting clinic serves a diverse population of pregnant women from an urban area, the study sample primarily consisted of highly educated women who agreed to participate. Previous research has suggested that a lower educational level is associated with a higher risk of low back pain [58, 59], but the association with PPGP remains unclear [38]. As women with higher educational levels may be more likely to participate in health research [60], the study sample may not fully represent women with different educational, socioeconomic, or demographic characteristics. Consequently, the generalizability of the findings should be interpreted with some caution. Future studies should aim to recruit participants with more heterogeneous educational and socioeconomic backgrounds.

The cross-sectional design allowed an initial description of satisfaction with individually tailored physiotherapy for PPGP and provided preliminary insights into factors associated with postpartum disability. However, causal relationships cannot be established, and longitudinal studies are needed to further investigate predictors of recovery and persistent symptoms.

## Conclusion

Individually tailored physiotherapy for PPGP in pregnancy was perceived as beneficial by most participants. However, a substantial proportion of women reported persistent symptoms four months postpartum, and many did not meet recommended levels of physical activity. Concern about pain was the only factor associated with disability levels, suggesting that psychological aspects of pain perception may play an important role in postpartum functioning. These findings highlight the potential value of addressing both physical and psychological aspects of PPGP and suggest that continued follow-up after pregnancy may be beneficial for women experiencing persistent symptoms. 

## Supplementary Information

Additional file 1.

## Data Availability

The datasets used and/or analysed during the current study are available from the corresponding author upon reasonable request.

## Abbreviations

BMI: Body Mass Index
GRC: Global Rating of Change
ICF: International Classification of Functioning, Disability and Health
NRS: Numeric Rating Scale
PPGP: Pregnancy-related Pelvic Girdle Pain
PGQ: Pelvic Girdle Questionnaire
SD: Standard Deviation
