# Supplementary material for: Individually tailored physiotherapy interventions for pregnancy-related pelvic girdle pain: functioning, physical activity, and treatment satisfaction four months postpartum. A cross-sectional study
Source: BMC Pregnancy Childbirth. 2026 Jul 7;26:723. doi: 10.1186/s12884-026-09617-w (PMC13340105; doi:10.1186/s12884-026-09617-w)
Supplement: Supplementary file 1 — Additional file 1. [file 12884_2026_9617_MOESM1_ESM.pdf]

GÖTEBORGS UNIVERSITET

Sahlgrenska academy

Institute of Neuroscience and Physiology

Section of Health and Rehabilitation

Unit of Physiotherapy

How does treatment according to national guidelines for pregnancy-related pelvic girdle pain meet women's needs for desired function in daily activities and work?

Questionnaire

Follow-up 4 months after childbirth

(The original questionnaire in Swedish was used in the study. It was translated to English before submission: initial draft by AI, and then checked manually for accuracy.)

Section A – Background

1. **What year were you born?** _________
2. **How tall are you?** _________ cm
3. **Your current weight:** _________ kg
4. **Have you been diagnosed with any medical conditions by healthcare professionals?**
   - ☐ No
   - ☐ Yes, if so, which? _____________________________
5. **How many pregnancies have you had?** _________
6. **How was your most recent childbirth?**
   - ☐ Vaginal delivery
   - ☐ Cesarean delivery
7. **Did you experience any complications during your most recent childbirth?**
   - ☐ No
   - ☐ Yes, if so, what kind? _____________________________
8. **Marital status:**
   - ☐ Single
   - ☐ Married/cohabiting
9. **What is your highest completed education?**
   - ☐ Elementary school
   - ☐ High school
   - ☐ College/university
10. **How well would you say you are managing financially?**

☐ Very well

☐ Quite well

☐ Neither well nor poorly

☐ Quite poorly

☐ Very poorly

11. **How would you summarize your current general health status on a scale from 0 to 10, where 0 is "Best possible condition" and 10 is "Worst possible condition"?** *(Circle your answer).*

    0 – 1 – 2 – 3 – 4 – 5 – 6 – 7 – 8 – 9 – 10

12. **How many days per week are you physically active at a moderate intensity for at least 30 minutes at a time?**
(Moderate intensity means activity that causes a slight increase in breathing or pulse rate, e.g., brisk walking, cycling, vacuuming, gardening.)
_________ days

13. **How many days per week are you physically active at a high intensity for at least 20 minutes at a time?**
(High intensity means activity that causes a significant increase in breathing or heart rate, e.g., running, aerobics, or heavy gardening.)
_________ days

14. **If you are not as physically active as you would like to be, what do you think is the reason for this? Please write any comments below.**

Section B – Pain and Discomfort

15. **If you have experienced muscle and joint pain in the past four weeks, please indicate on the diagram where you have had pain.**

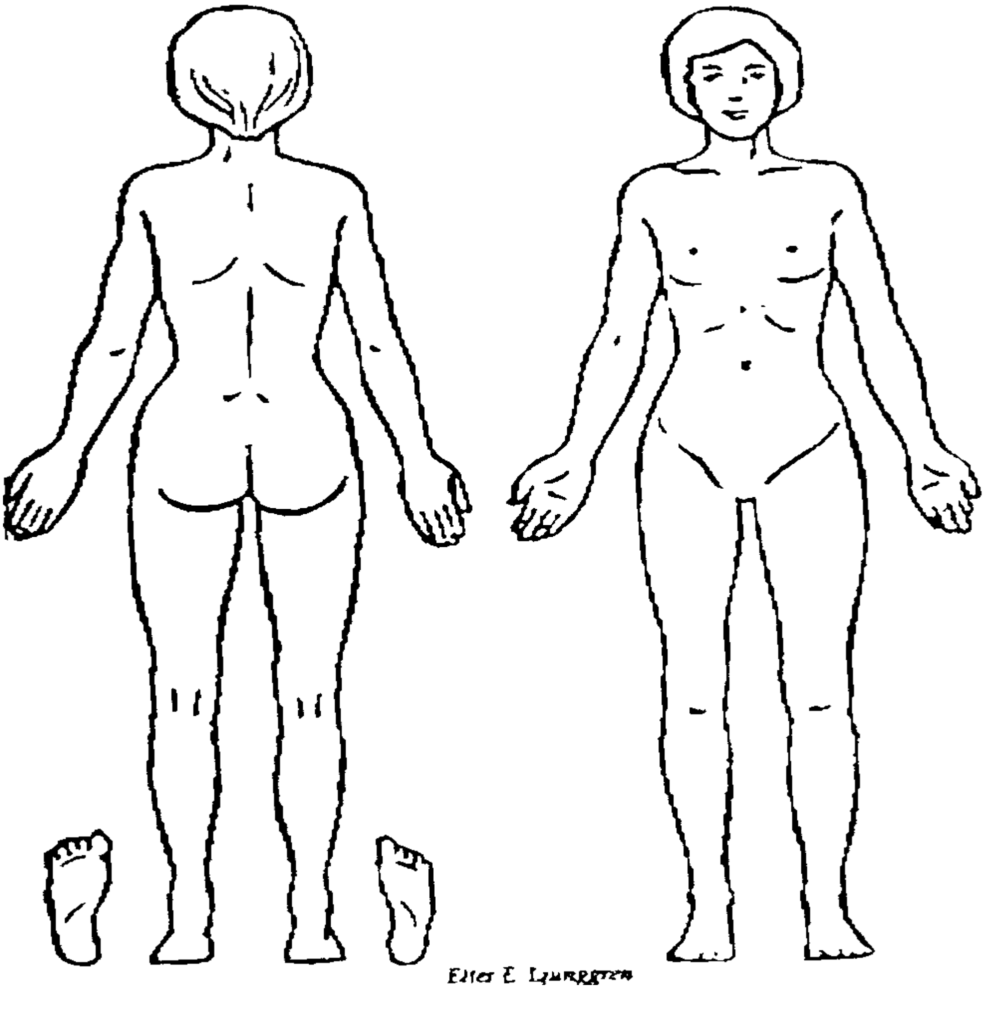

16. **In the past four weeks, have you experienced pain in the lower back/pelvic girdle (in the areas marked in the figure)?**
Do not report pain you had due to fever.

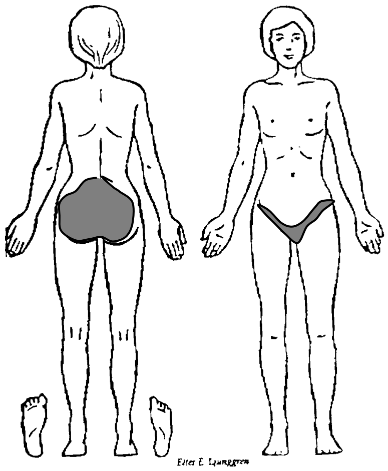

☐ Yes

☐ No

17. **If you have experienced lower back or pelvic girdle pain, was the pain severe enough to limit your usual activities or change your daily routine for more than a day?**

☐ Yes

☐ No

18. **If you have experienced lower back or pelvic girdle pain, how often have you had the pain?**

☐ Some days

☐ Most days

☐ Every day

19. **If you have experienced lower back or pelvic girdle pain, have you had this pain before pregnancy?**

☐ Yes

☐ No

20. **On a scale from 0 to 10, where 0 is "No pain" and 10 is "Worst imaginable pain", how strong was your pain in the past 48 hours on average?** *(Circle your answer.*)

0 – 1 – 2 – 3 – 4 – 5 – 6 – 7 – 8 – 9 – 10

21. **On a scale from 0 to 10, where 0 is "No pain" and 10 is "Worst imaginable pain", how strong was your pain in the past 48 hours on average?** *(Circle your answer.)*

0 – 1 – 2 – 3 – 4 – 5 – 6 – 7 – 8 – 9 – 10

22. **On a scale from 0 to 10, where 0 is "Not worried at all" and 10 is "Extremely worried", how much does your pain concern you?** *(Circle your answer.)*

0 – 1 – 2 – 3 – 4 – 5 – 6 – 7 – 8 – 9 – 10

23. **Are you taking any pain medication for your lower back- or pelvic pain?**

☐ Yes

☐ No

PGQ – Pelvic Girdle Questionnaire:

To what extent do you find it problematic to carry out the activities listed below because of pelvic girdle pain? For each activity tick the box that best describes how you are today

**How problematic is it for you because of your pelvic girdle pain to:**	**Not at all (0)**	**To a small extent (1)**	**To some extent (2)**	**To a large extent (3)**	
Dress yourself	☐	☐	☐	☐	
Stand for less than 10 minutes	☐	☐	☐	☐	
Stand for more than 60 minutes	☐	☐	☐	☐	
Bend down	☐	☐	☐	☐	
Sit for less than 10 minutes	☐	☐	☐	☐	
Sit for more than 60 minutes	☐	☐	☐	☐	
Walk for less than 10 minutes	☐	☐	☐	☐	
Walk for more than 10 minutes	☐	☐	☐	☐	
Climb stairs	☐	☐	☐	☐	
Do housework	☐	☐	☐	☐	
Carry light objects	☐	☐	☐	☐	
Lift heavy objects	☐	☐	☐	☐	
Get up/sit down	☐	☐	☐	☐	
Push a shopping cart	☐	☐	☐	☐	
Run	☐	☐	☐	☐	
Carry out in sporting activities*	☐	☐	☐	☐	☐
Lie down	☐	☐	☐	☐	
Roll over in bed	☐	☐	☐	☐	
Have a normal sex life*	☐	☐	☐	☐	☐
Push something with one foot	☐	☐	☐	☐	

(If not applicable, mark box on the right.)*

**How much pain do you experience:**	**None (0)**	**Some (1)**	**Moderate (2)**	**Considerable (3)**
In the morning	☐	☐	☐	☐
In the evening	☐	☐	☐	☐

**To what extent because of pelvic girdle pain:**	**Not at all (0)**	**To a small extent (1)**	**To some extent (2)**	**To a large extent (3)**
Has your leg/have your legs given way?	☐	☐	☐	☐
Do you do things more slowly?	☐	☐	☐	☐
Is your sleep interrupted?	☐	☐	☐	☐

Section C – Treatment

24. **On a scale from 0 to 10, where 0 is "Very much worse " and 10 is "Completely recovered", regarding your pelvic girdle pain, how would you describe yourself now compared to when you were in contact with a physiotherapist at Närhälsan Eriksberg during your most recent pregnancy?** *(Circle your answer.)*

    0 - 1 – 2 – 3 – 4 – 5 – 6 – 7 – 8 – 9 – 10

25. **Did the treatment you received meet your expectations?**

☐ Yes

☐ No

26. **Have you experienced any side effects from the treatment you received for pelvic girdle pain during your most recent pregnancy?**

☐ No

☐ Yes, if so, what? ___________________________

27. **How satisfied are you with the treatment you received for your pelvic girdle pain on a scale from 0 to 10, where 0 is "Not satisfied at all" and 10 is "Completely satisfied"?** *(Circle your answer.)*

    0 – 1 – 2 – 3 – 4 – 5 – 6 – 7 – 8 – 9 – 10

28. **Is there anything you feel was missing from the treatment you received for your pelvic girdle pain? Please write any comments below.**
